# Environmental audit scoring evaluation: evolution of an evidence-based environmental assessment tool to support person-centered care

**DOI:** 10.3389/frdem.2024.1470036

**Published:** 2024-11-18

**Authors:** Robert A. Wrublowsky, Migette L. Kaup, Margaret P. Calkins

**Affiliations:** ^1^RAW Consultants, Winnipeg, MB, Canada; ^2^Department of Interior Architecture & Industrial Design, College of Architecture Planning and Design, Kansas State University, Manhattan, KS, United States; ^3^IDEAS Institute, Cleveland Heights, OH, United States

**Keywords:** nursing homes, assisted living, architectural design, person-centered care, dementia, environmental assessment, long term care, design guidelines

## Abstract

Long-term care settings are at the center of strongly debated approaches to policies that shape the delivery of care and operational practices. There is advocacy for transformational change within these settings to support a person-centered approach to care delivery, but it is difficult and multifaceted involving everything from changing the level of staffing and care models to developing appropriate metrics to assess an individual's quality of life. The physical environment is a key component for accomplishing the organizational and operational goals related to person-centered care, but providers and their design teams need the appropriate tools to guide evidence-based decision-making. The Environmental Audit Scoring Evaluation (EASE) is a tool that helps lend structure to the process of developing the environment for our senior population—especially those living with dementia. This perspective article will discuss how EASE aims to align the design process to more fully support the myriad environmental elements that have a demonstrable impact on the individual, and the associated quality of life they experience. The article will also explore how EASE differs from previous planning strategies that did not prioritize residents' psychological wellbeing in conforming to current person-centered philosophies.

## Introduction

It is widely recognized that the designed environment impacts—both negatively and positively—individuals living with dementia (Calkins et al., 2024). Literature reviews on this topic identify a growing number of studies that examine the impact of specific environmental features or characteristics on outcomes, such as engagement, falls, cognition and more (Chaudhury et al., 2017; Marquardt et al., 2014). This research has largely run parallel with significant changes in the model of care in long-term care, from a staff-centric, medical model to person-centered or person/self-directed models (Koren, 2010). Person-Centered Care (PCC) origins date to over 75 years ago through the work of Rogers (1946) and then later broadened by Kitwood (1998) who, had a major influence in the field of dementia care through his pioneering work that provides a theoretical basis for delivering person-centered care for people with dementia. PCC is the essence that respects and appreciates each person as a unique, valuable individual that places the person at the center of their own care through shared decision-making, equality of communication, and mutual respect (Mitchell and Agnelli, 2015).

PCC is generally accompanied by environmental changes that seek to enable these objectives by making shared residential care settings more like a home than a hospital, complete with a residential front door, functional kitchen, dining, and living rooms as well as a higher percentage of private bedrooms with ensuite, three-fixture bathrooms (Brouwers et al., 2023; Calkins et al., 2024; Meyer, 2023). However, what is not known is how the constellation of environmental features and characteristics typically included in these household models impacts residents living with dementia. This is in large part because the majority of existing environmental assessment tools, particularly those used in North America, were created prior to the development of household models, and thus don't adequately differentiate between settings on these important features. It is regularly acknowledged, however, that to accomplish person-centered and dementia-supportive care practices, the role of the physical environment must be considered (add citations).

## Theoretical foundations

Theoretically, the Competence Press model developed by Lawton and Nahemow (1973) is one of the most commonly cited frameworks that describe the relationship between people, who are viewed as having a set of competencies, and the environment, which is viewed as exerting press or demands on an individual. When these are in balance, an individual is at their adaptation level, with minor changes in press allowing the individual to remain in a state of positive affect and adaptive behavior, while more significant changes in press, either higher or lower, move an individual into a state of negative affect and maladaptive behavior. While Lawton and Nahemow define and identify assessments for competencies across several domains (biological health, sensorimotor functioning, cognitive skill, etc.) they are less specific on the environmental side of the model and focus primarily on the amount of press. Calkins (Forthcoming), Building on the work of Carp (1976), has postulated that when an environment is designed to support the individual living with dementia, it effectively serves to increase the functional level—or competency—of that individual. With the appropriate environmental support, individuals with dementia can engage effectively with more and a wider range of environmental press.

This is in line with the work of early researchers who conceptualized various frameworks or sets of therapeutic goals that outlined what would be supportive for people living with dementia in shared residential settings (Calkins, 1988; Calkins et al., 2024; Cohen and Weisman, 1991; Fleming and Bowles, 1987; Regnier, 1992; Zeisel et al., 1994). Much of this work was conducted by environmental gerontologists and thus the designed environment was integral to the movement for changing the paradigm of care from the beginning. This led to the development of several design guides, the first generations of which could only hypothesize about the relationship between environmental features and resident outcomes, due to the limited research that had been conducted at that point in time. While the overarching goal was to enhance the quality of life and wellbeing of individuals living with dementia, making the link between specific environmental features and specific outcomes of interest was tenuous at best.

## Design practices embrace a research agenda

After three decades of research on how the designed environment impacts individuals living with dementia in shared residential settings, there was sufficient evidence to create a design guide that would more specifically postulate links between environmental features and characteristics to specific outcomes of interest such as cognition, functioning, and behaviors. A practicing architect, Robert Wrublowsky of MMP Architects, in Winnipeg, Canada was actively engaged with the province's initiatives for addressing the need for skilled nursing care centers (referred to as personal care homes). Recognizing that existing Canadian design standards lagged behind person-centered care and dementia-supportive strategies, Wrublowsky petitioned the Winnipeg Regional Health Authority to commission him to develop a new evidence-based set of comprehensive guidelines that were person-centered and dementia-supportive.

**Figure 1:**
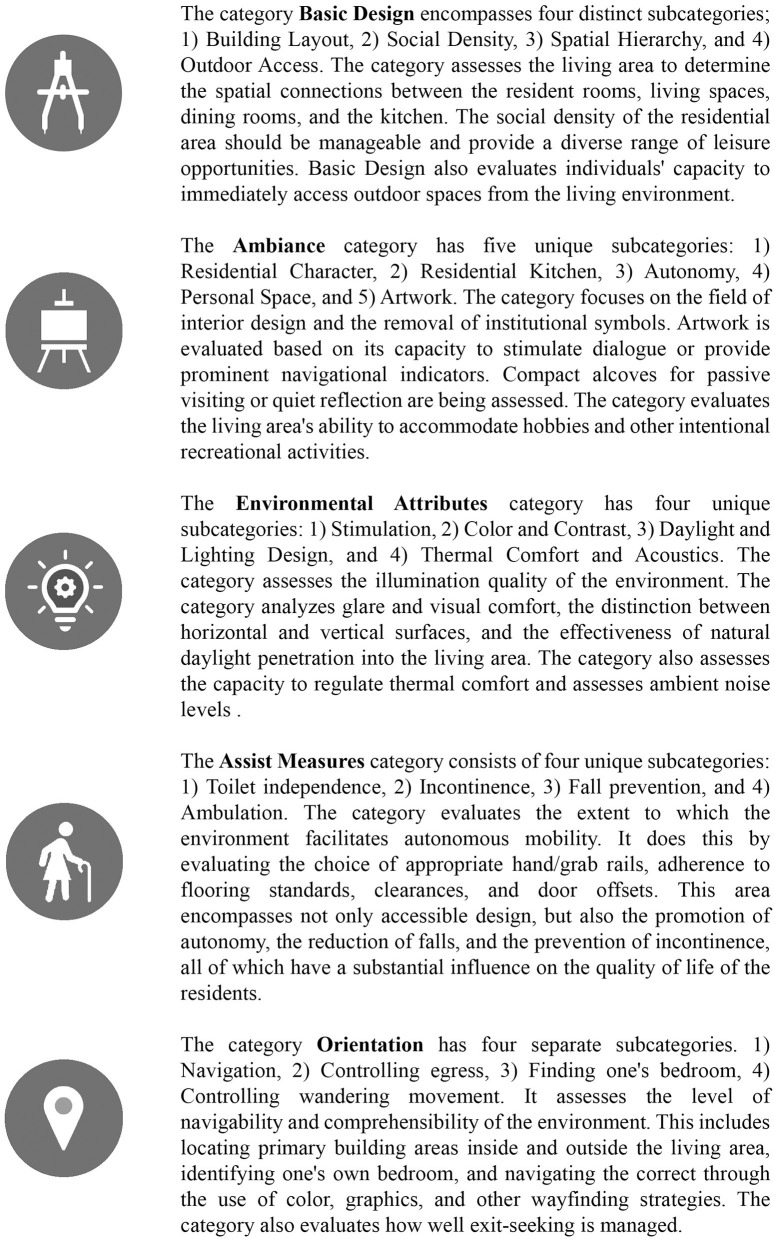
Five categories for planning strategies to support person-centered care experience.

The resulting work, *Design Guide for Long Term Care Homes* (Wrublowsky, 2018) was primarily based on a literature review conducted by Marquardt et al. (2014) that identified relationships between design and user experiences and impacts. The initial *Design Guide* (Wrublowsky, 2018) framed planning strategies around five categories; basic design attributes, ambiance, environmental attributes, assistive measures to support independence, and orientation (see Figure 1). The design interventions provided in the guide were specific and referenced research that supported its application. The Environmental Audit Scoring Evaluation (EASE) instrument evolved from this guideline.

Typical jurisdictional and national design guidelines primarily focus on safety and risk-averse solutions. EASE includes items that assess safety factors but go beyond these prescriptive standards and identify interventions that are more closely aligned with person-centered care values. The initial goal of developing the EASE tool was to collect research-backed design strategies that speak to interventions that are not typically part of a more prescriptive set of standards that focus on safety, security, and infection control. Wrublowsky developed the first version of the tool to assist a private long-term care operator in Alberta, Canada, to examine more closely how they could improve their environments to be more responsive to person-centered care strategies by assessing 164 unique items. Seven care communities were evaluated using the early version of the tool and administrators were provided with explanations on the efficacy of physical design characteristics to influence certain behaviors. This early version of the tool had variable response structures that included both dichotomous and Likert-type scoring.

## Design strategies become research

As Wrublowsky disseminated the outcomes of his work in Canada, the utility of the tool gained the attention of Dr. Margaret Calkins, an established researcher and environmental gerontologist. Together Wrublowsky and Calkins revised the tool for consistent 5-point scoring that reflected a graduated spectrum of design features from medical model to household model Settings. This updated version of the tool underwent face validity with a review from 22 subject matter experts who provided feedback and verified the value of each scoring criterion to the delivery of person-centered care (Kaup et al., 2023).

After these revisions, the EASE comprised 147 items with a consistent scoring algorithm (all items scored on a 5-point scale). The structure continued to be based on hypothesized relationships between items (environmental characteristics or features) and resident and staff outcomes. EASE includes many design strategies that are often overlooked when designing long-term care environments. EASE incorporates both activities of daily living and many instrumental activities of daily living (IADL) design interventions that support a person to dress independently, enhance the quality of family engagement and visitation, make it easier to do their laundry if they are capable, and incorporate relational dining strategies that promote flexible mealtimes and support for meal preparation. EASE also addresses an environment that supports staff and care providers' roles while making it easier for them to be as much of a companion to residents as they are clinicians (a goal of person-centered care).

This version was subsequently assessed for reliability, validity, and capacity to distinguish between different environmental settings by evaluating 28 living areas within 13 different skilled care communities (Kaup et al., 2023). All participating care communities in this study were adopters of person-centered care practices but varied in the type of environments where care was delivered. Results demonstrated that the EASE was able to distinguish between more medical-based (traditional) models and household models. This study also provided evidence that the EASE tool could be applied in non-Canadian environments and was effective in both dementia-specific settings (e.g., special care units) and dementia-inclusive living areas.

Establishing the link between different constellations of environmental elements and outcomes of interest is a critical next step in the development of this tool. Refining the factorial structure of the EASE tool is underway. The next version of the tool is being administered to a broader sample of over 225 living areas from over 100 providers across the US and Canada. The goal is for the EASE to be used to identify constellations of factors, such as an environment that reflects more elements of home rather than an institution, which might be associated with resident goals such as positive wellbeing, functional independence, and social engagement as well as staff and organizational outcomes (e.g., reduced turnover, staff satisfaction, etc.).

Using the EASE tool in multiple settings allows for further refinement of the scoring criteria to reflect the intent of the design feature related to supporting a person-centered outcome or autonomy for residents. EASE is intended to pick up where other design resources end. It establishes the known relationships of design interventions to reduce environmental pressure triggering greater agitation and social withdrawal. EASE identifies environmental strategies associated with positive outcomes that contribute to a person's psychological needs. To achieve outcomes of positive wellbeing through a cultural change in an organization there must be three types of transformation: personal, physical, and operational (Power, 2017). EASE focuses on developing the physical aspects of the environment to support a person's wellbeing and culture change.

**Figure 2:**
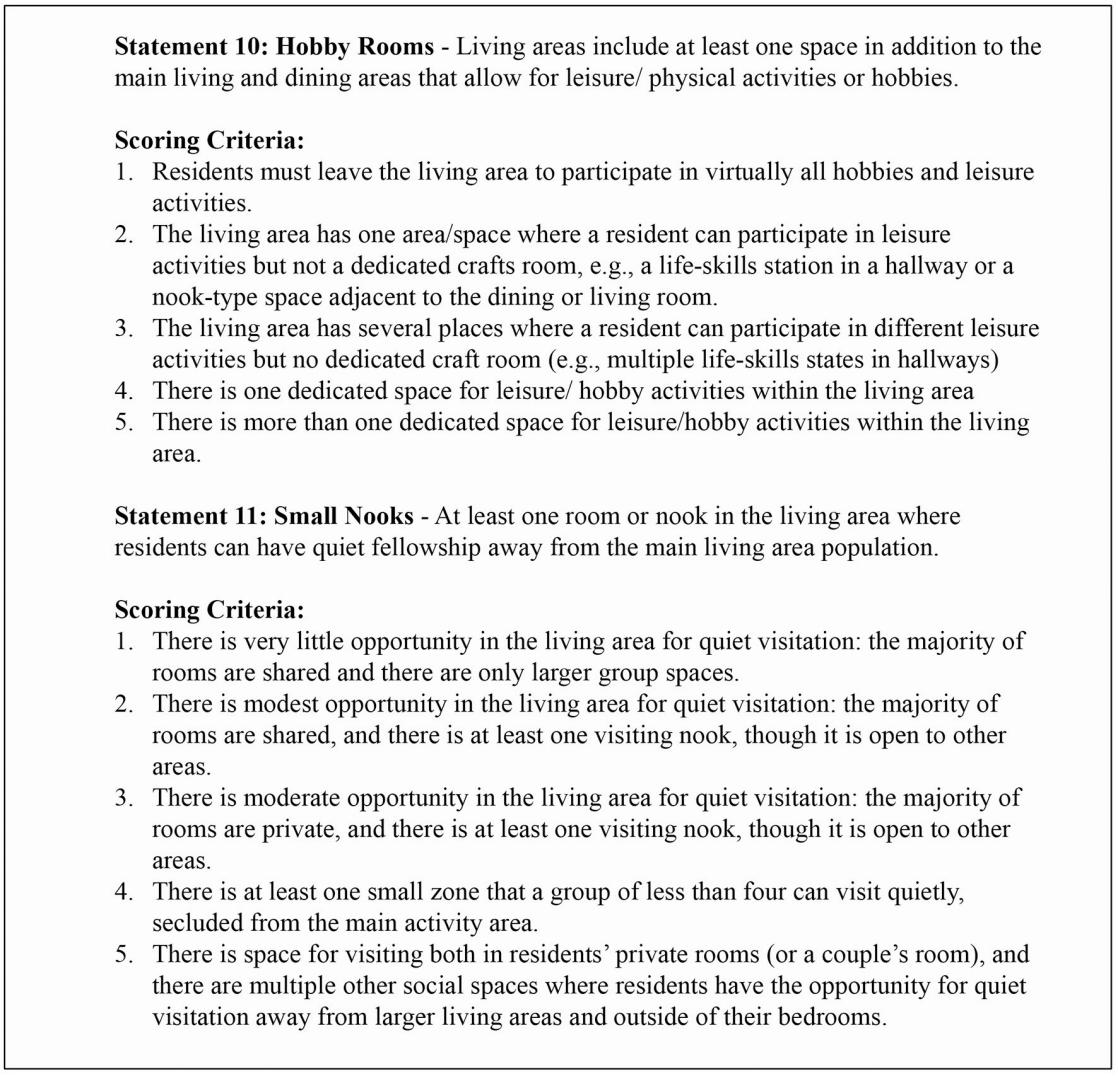
Examples of statements and scoring criteria.

The design strategies also include environmental features to support those who have higher acuity by improving sleep hygiene, nutrition intake, and cognition. EASE aims to identify environmental elements that help maintain a person's independence to the extent of their capacity, including positive ambulation support, sit-to-stand mechanics, and fall reduction. Again, theoretically, this should effectively serve to increase the functional levels—or competencies—of individuals living with dementia. People living with dementia may become withdrawn because of the symptoms of the disease, but individuals may also withdraw because they are not provided with a variety of leisure experiences that allow them to continue to experience joy, purpose, and engagement (Dupuis et al., 2012). EASE responds by including two statements under the Category of Basic Design, which recognizes the importance of the social density of the environment and its ability to provide people with purposeful leisure spaces (see Figure 2).

Further areas of research topics evaluated through EASE include light exposure. Research has consistently shown that increased light exposure can significantly improve cognitive function. For instance, seasonal affective disorder can be alleviated after about a week of daily light exposure, typically at a level of 2,500 lux for 2 h (Van Someren and Riemersma-Van Der Lek, 2007). Historically, studies have shown that the average healthy person is exposed to more than 1,000 lux with an average daily exposure of 1.7 h compared to an elderly institutionalized person who is exposed to a median light level of only 54 lux and was exposed to more than 1,000 lux for only 10.5 min per day [Van Someran et al. (2002), p. 215]. Based on the data collected in over 200 nursing homes (circa 2021–2024) using the EASE tool, median light levels and daylight exposure are still far below these recommended levels in many institutional settings. If designers use the EASE tool as a planning guide, they will be able to program living areas in nursing homes to locate socialization spaces with direct access to windows and natural daylight exposure. EASE responds to this by including a statement under the Category Environmental Attributes for Access to Sunlight (Statement 72) that awards the highest points to environments that locate the main socialization areas within the living area on an outside wall and have direct access to natural daylight, thus supporting a natural circadian rhythm.

## The importance of measuring the role of the built environment

The projected growth of millions of individuals who will live with Alzheimer's and other types of dementia and neurocognitive disorders in the next 25 years (Alzheimer's Association, 2024; World Health Organization, 2023) demonstrates the importance of approaching therapeutic practices from a holistic perspective. The mounting evidence that the design of the built environment influences and impacts users of skilled care, especially those living with dementia, substantiates the need for environmental measurement tools with the capacity to connect these distinguishable environmental characteristics with different outcomes of interest.

Numerous authors (e.g., Duan et al., 2020, p. 220; Miller et al., 2014, p. 1676; Miller et al., 2018; Lima et al., 2020, p. 8; Brownie and Nancarrow, 2013, p. 9) have identified the persistent challenge in correlating the effect of delivery care strategies, such as person-centered care and household model environments of care, to measurable outcomes and impacts, both for quality of care and quality of life. The prevailing challenge in linking outcomes to delivery models of care is the lack of a consistent and reliable measure for articulating the environment of care that is not consistent from setting to setting. While the environment is often cited as an important component of creating a PCC environment, its dimensions have not been studied or articulated with the same scrutiny as care or workplace practices but rather they are discussed generically and in heuristic terms (e.g. Hill et al., 2011; Miller et al., 2018, p. 989). There are also inconsistencies in the measure of the application (or “dose”) of person-centeredness in the delivery of care, and there has been no tool to articulate the distinctions between environmental types. It is worth noting that there are a number of tools designed to evaluate long-term care environments. A systematic review by Calkins et al. (2022) compares 13 different environmental assessment tools for dementia long-term care settings, developed between 2001 and 2017. Many of these were developed prior to the evolution of the household model, and thus are of limited value when trying to systematically compare traditional and household settings. Of the tools reviewed, the Environmental Audit Tool (EAT) developed in Australia (Fleming, 2011) is the only tool that addresses the household model. However, the EAT employs primarily dichotomous scoring (present, not present), which limits the ability to articulate subtle differences, and many elements of household design are not addressed. The authors (Calkins et al., 2022) conclude that there is a need for environmental assessment tools that address the full spectrum of environmental features embedded in household models that can be described with a fine level of detail to capture subtle differences between settings.

The EASE tool is a significant advancement in the capacity to clearly and succinctly articulate the physical characteristics of the environment of care. This tool is built on the evidence of those documentable features that have been shown to have an impact on care and workplace practices (Hartmann et al., 2013). EASE is specifically inclusive of age-appropriate, dementia-supportive, and person-centered care features. These characteristics are often the basis of the household model settings but can be effectively incorporated into remodeling efforts of more traditional, medical-style nursing homes. These environmental features also represent nonpharmacological support for persons with dementia.

As testing of the EASE tool has advanced, the operational and organizational variables that are critical to these measurements are coming into focus. The environment of care that acts as the surround for daily routines can comprise diverse combinations of characteristics that contribute to or distract from a dementia-inclusive-homelike setting. The unique structure of the EASE tool manages the complexity of these planned care settings by measuring these characteristics and placing each living area on a spectrum relative to supportive and person-centered living. The items are based on the established needs of residents and staff as well as the organizational and operational factors that contribute to how the environmental features are used.

Another unique aspect of this research is the focus on the primary area where a resident is likely to spend most of their day. This defines the unit of analysis as a living area; an identifiable portion of a nursing or care home where a group of residents share some common spaces (e.g., a hallway, living room, dining room, etc.). The EASE tool provides a discrete measurement that captures the strengths or weaknesses of the environment that surrounds the residents. The tool's capacity to distinguish household settings from more institutional living areas demonstrates its validity in measuring the stated design features focusing on environmental traits that prioritize residential life above institutional routines and clinical architecture. This is a critical dimension to the tool's utility as many existing nursing homes that are currently more institutional in their design can still effectively be remodeled by focusing on those environmental changes that have the biggest impact on person-centered care strategies.

As previously noted, the quality of environmental research for senior care settings for people with dementia has been impeded by the lack of high-quality, comprehensive, objective assessment tools that are both descriptive and quantitative. The EASE tool represents the next generation of assessment instruments to fill this gap. It also differentiates between medical model design features and ones that reflect person-centered care values and household design (Kaup et al., 2023). The significance of this work is the ability to use the EASE to enhance understanding of the role of the built environment on outcomes for residents and long-term care staff.

Investments in better care also means utilizing all the resources a community has to work with including programs, services, staff, and the setting. Carefully planning infrastructure is critical and making the changes that will matter the most is the best way to get a return on that investment. The EASE tool has been specifically designed by professionals knowledgeable about evidence-based planning and design, with input from people who live and work in skilled care settings. EASE is structured to have versatility for users; it can be applied as an evaluative tool in existing buildings to articulate features that are/are not supportive of PCC values and then be used to target limited remodeling dollars. It can also be used in new construction as a programming and design instrument to communicate priorities. This makes evidence-based information accessible to a cross-section of professionals who can use this information to implement quality improvements. Its nuanced approach to scoring gives providers multiple layers of potential interventions with varying costs (e.g., from changing light bulbs to increase foot-candle levels to investing in lighting with tuneable LED systems).

## The future of evidence-based dementia-inclusive design

The development of this instrument is ongoing, and the next planned stage of evaluative research will add evidence to the correlations between constellations of design features and outcomes in quality of care (e.g., MDS metrics) and quality of life. A valuable repository of evidence-based information can be created with a valid, scaled tool that can ultimately be used to explore potential relationships between environmental elements and identifiable and measurable benefits for residents and staff. The significance of this ongoing work is that this tool has the potential to fill a known gap in the research of quality measures for person-centered care and dementia-inclusive practices as well as the design strategies that are employed to create these settings. The EASE does not propose that single environmental elements will impact, in a measurable way, these associated benefits, but that a constellation of factors, such as an environment that reflects more elements of home rather than an institution, might be associated with resident outcomes such as positive wellbeing, functional independence, and greater social engagement. Linking these experiences with design strategies will significantly advance the research on evidence-based design approaches that contribute to therapeutic outcomes for individuals who live in long-term care settings.

EASE helps facilitate positive first steps to modifying the built environment once a commitment to Person-Centered Care reform has begun. Starting with the realization that institutional care models do not promote a high quality of life, many long-term care providers are beginning to modify their policies to prioritize resident choice—a core value of person-centered care. It has been asserted that even providers who suggest they are resident-centered still maintain policies that are at odds with residents' positive lived experiences (Koren, 2010). Additionally, as mentioned above, codes and regulations have traditionally had a deep focus on safety and clinical outcomes largely to the exclusion of the quality of residents' everyday lived experiences and wellbeing. Also, while they are couched as being minimum standards, they often become the default maximum standard—meaning the system is geared to maintaining the lowest standards required. Both jurisdictional authorities and care providers must accept that this is not sufficient and that adopting person-centered care values, practices, and environments will enhance wellbeing and quality outcomes for residents, enhance staff experiences, and increase family satisfaction, all of which better support organizational outcomes. For organizations seeking to more deeply adopt person-centered values and practices to maximize residents' wellbeing, quality of life as well as the quality of care, it is essential for the organization's values to drive person-centered policies and practices and be supported by an environment that reflects those same values. Being derived from person-centered values and household models, the EASE can help facilitate an organization's adoption of a person-centered care setting, but it must also align with person-centered values, policies, procedures and training, and a regulatory process that shares these same values.

## Data Availability

The original contributions presented in the study are included in the article/supplementary material, further inquiries can be directed to the corresponding author.
